# ALOX5 acts as a key role in regulating the immune microenvironment in intrahepatic cholangiocarcinoma, recruiting tumor-associated macrophages through PI3K pathway

**DOI:** 10.1186/s12967-023-04804-1

**Published:** 2023-12-20

**Authors:** Jialu Chen, Yue Tang, Delong Qin, Xiaopeng Yu, Huanjun Tong, Chengwei Tang, Zhaohui Tang

**Affiliations:** 1 Department of General Surgery, Xinhua Hospital, Shanghai Jiao Tong University, School of Medicine, Shanghai, 200092 China; 2 Department of Blood Transfusion, Xinhua Hospital, Shanghai Jiao Tong University, School of Medicine, Shanghai, 200092 China; 3 Shanghai Key Laboratory of Biliary Tract Disease Research, Xinhua Hospital, Shanghai Jiao Tong University School of Medicine, Shanghai, 200092 China

**Keywords:** Intrahepatic cholangiocarcinoma, Tumor-associated macrophages, Tumor microenvironment, Lipid metabolism, Single-cell analysis

## Abstract

**Background:**

Intrahepatic cholangiocarcinoma (ICC) is poorly treated due to the presence of an inhibitory immune microenvironment. Tumor-associated macrophages (TAM) are an important component of TME. ALOX5 is an important lipid metabolism enzyme in cancer progression, but the mechanism by which it regulates TAM to promote ICC progression is unknown. The aim of this study was to investigate the potential mechanism of TAM regulation by ALOX5 and the translational effect of targeting ALOX5.

**Methods:**

In this study, we investigated the association between the spatial localization of epithelial cells and TAMs by combining scRNA-seq analysis with multiplex immunofluorescence analysis. Through bulk sequencing analysis and spatial analysis, lipid metabolism genes closely related to TAM infiltration were screened. In vitro co-culture model was constructed to verify that ALOX5 and its downstream metabolite LTB4 promote M2 macrophage migration. Bulk sequencing after co-culture combined with single-cell analysis was performed to identify key pathways for up-regulation of M2 macrophage migration. Finally, the effect of CSF1R inhibitor (PLX3397) combined with ALOX5 inhibitor (Zileuton) in vivo was investigated by by xenograft tumor formation experiment in nude mice.

**Results:**

ALOX5 in ICC cells was a key lipid metabolism gene affecting the infiltration of M2 macrophages in TME. Mechanically, LTB4, a metabolite downstream of ALOX5, recruited M2 macrophages to migrate around tumor cells by binding to BLT1/BLT2 and activating the PI3K pathway, which ultimately lead to the promotion of ICC progression. Targeting CSF1R in combination with ALOX5 inhibitor effectively reduced tumor volume and M2 macrophage infiltration abundance.

**Conclusion:**

In ICC, LTB4, a metabolite secreted by ALOX5 of epithelial cells, binded to BLT1/BLT2 on TAM surface to activate PI3K pathway and promote TAM migration, thus promoting ICC progression. Targeting CSF1R in combination with ALOX5 inhibitor for ICC is a promising combination therapy modality.

**Supplementary Information:**

The online version contains supplementary material available at 10.1186/s12967-023-04804-1.

## Introduction

Intrahepatic cholangiocarcinoma (ICC) is the second most common primary liver malignancy, accounting for about 20% of all liver malignancies [1]. The morbidity and mortality of ICC are still on the rise worldwide. Radical resection is the only possible cure for ICC. However, most patients are found to be advanced, and their 5-year survival rate is often less than 5–10% [2]. Due to the high heterogeneity of pathological characteristics and gene expression profile, the effect of radiotherapy and chemotherapy is extremely unsatisfactory in ICC [3]. Although targeted therapy is less toxic, targeting specific genes is not effective in killing tumor cells in most cases [4]. In recent years, immunotherapy has shown great potential in anti-tumor therapeutic applications [5–7]. However, in immune "cold" tumors such as ICC, the effect of immunotherapy is often very poor. With the development of immunotherapy, it is found that tumor microenvironment (TME) is an important factor affecting the therapeutic effect [8].

Alterations in lipid metabolism are one of the most important features of tumor metabolic reprogramming [9, 10]. Increased lipid synthesis or uptake promotes ICC cell proliferation. Also the synthesis of more complex lipid substances increases, acting as mediators for various biological pathways [11]. ALOX5 (5-lipoxygenase, 5-LO) is an enzyme responsible for the synthesis of a series of biologically active lipid signaling molecules, catalyzing arachidonic acid to produce metabolites such as 5-hydroxydodecapentaenoic acid (5-HETE) and leukotrienes (LTB4, etc.), which have been shown to be lipid mediators of inflammation in various diseases, including cancer. This lipid mediator is associated with tumorigenesis and progression, but the exact mechanism is not clear [12, 13]. At present, the research on ICC metabolic reprogramming is often limited to tumor cells, while the real tumor growth environment is inseparable from TME, and few studies have been conducted on metabolic reprogramming of TME. TME is a major contributor to the metabolic ecology of tumors. Further study of metabolic interactions between different cells in TME will help to fundamentally improve the therapeutic translational potential of targeting metabolic pathways. A better understanding of different metabolic dependencies and interactions between tumors and immune or stromal cells may provide a unique opportunity for metabolic therapy.

Tumor-associated macrophages (TAMs) are a kind of plastic immune cells, which are the most abundant immune cells in the immune microenvironment of ICC, especially in the front of tumor invasion and perivascular sites [14]. TAMs can release many cytokines and interact with ICC cells and other stromal cells to create a favorable environment for ICC growth [15]. TAMs are often divided into two different states: classical activation (M1 type) and alternate activation (M2 type) macrophages [10, 16]. It is generally believed that TAMs are more inclined to M2 phenotype [17, 18]. However, with the development of single-cell technology, the distinction between the two subtypes of TAMs is becoming more and more blurred. Current studies have focused more on how TAMs regulate the malignant phenotype of ICC cells [19–21]. However, there are few reports on how ICC cells interact with TAMs to form TME, which is beneficial to tumor growth.

In this study, we comprehensively explored the relationship between epithelial cells and TAMs of ICC in the TME by exploring published scRNA-seq data, combined with spatial localization analysis. Subsequently, we screened ALOX5, a lipid metabolism-related gene associated with TAM infiltration, by combining second-generation sequencing analysis. We validated the specific mechanism by which upregulated ALOX5 in ICC cells promotes M2-TAM infiltration in vivo and in vitro. We also explored the clinical translational significance of targeting TAMs in combination with ALOX5 inhibitors in ICC. From the perspective of tumor metabolic reprogramming regulating TME, our study provides insights into new therapies for malignant tumors using lipid metabolism drugs in combination with immunotherapy drugs. This may help to develop rational combination therapy strategies to overcome the adverse effects of TME on drug efficacy.

## Materials and methods

### Single cell sequencing analysis

The scRNA-seq data of ICC tissue and paracancerous tissue were obtained from the GEO website (GSE 138709). The dataset consisted of five ICC samples and three paracancerous samples [22]. We used the seurat package in R software for data preprocessing and analysis. We retained cells with nFeature > 300, nCount > 1000, and mitochondrial fraction < 10% & erythrocyte fraction < 3% for further analysis. We integrated cells from different samples using the harmony R software package, followed by dimensionality reduction clustering using the FindNeighbors and FindClusters functions (resolution = 0.5, select the first 15 PCs). Finally, nonlinear dimensionality reduction is performed using the Seurat "RunUMAP" function. Cell subpopulations were automatically annotated using the singleR package followed by manual annotation based on published studies. The strength of communication between cell subpopulations was inferred and visualized using the CellChat R package [23].

### Bulk RNA sequencing analysis

We collected 7 pairs of fresh surgically resected ICC samples and performed second-generation sequencing. Using Deseq2R package, the original count data were analyzed and 5754 differential genes were identified (pvalue < 0.05&|log2FC|> 1). We also downloaded FPKM data from the TCGA-CHOL dataset from the TCGA website, including 36 cancer tissues and 8 adjacent tissues. Immunoinfiltration analysis was performed on 36 cancer tissues based on the ssGSEA algorithm provided in R package-GSVA [1.46.0] [24], followed by correlation analysis with lipid metabolism genes. Enrichment analysis is implemented using the Clusterprofiler package.

### Immunofluorescence and immunohistochemical analysis

In this study, multiplex immunofluorescence staining was used to investigate the localization and interaction of multiple proteins in cells. Freshly excised ICC tissues were fixed with 4% paraformaldehyde and sectioned, followed by treatment with 0.1% Triton X-100. After blocking non-specific binding sites with 5% BSA, appropriate in situ antibodies were added to the sections, incubated for a period of time, and cells were washed with PBS. The above steps are repeated using antibodies from different species and appropriate fluorescent labels to stain different target proteins. The processed sections were placed on glass slides and processed with the appropriate mounting media. The staining results were observed using a fluorescence microscope and image acquisition was performed. The immunohistochemical procedure was similar to that described above, with sections incubated in ALOX5 primary antibody (proteintech, 10,021–1-Ig, 1:500) followed by secondary antibody, followed by dehydration and mounting.

### Cell culture and compounds

The cell lines H69, RBE, HCCC-9810, QBC-939, CCLP-1, HUCCT-1, THP-1 and RAW264.7 used in this study were obtained from the cell bank of Shanghai Institute of Biological Sciences. RAW264.7 cells were cultured in Dulbecco's Modified Eagle's Medium supplemented with 10% FBS, 1% penicillin streptomycin. Other cells were cultured in RMPI-1640 medium supplemented with 10% FBS, 1% penicillin streptomycin in a humidified incubator at 37 °C with 5% CO2. Zileuton, PLX3397, U75302, LY255283, Pictilisib, Rapamycin, MK-2206 were obtained from Selleckchem. PMA was obtained from Sigma and IL-4, IL-13 were obtained from Yeason. All compounds were reconstituted according to the manufacturer's instructions and stored as frozen stock solutions at − 20 °C. THP-1 cells were plated at 10^6 in 6 cm culture dishes, and stimulated with 200 ng/ml PMA for 12 h, then IL-4 and IL-13 were added at 20 ng/ml, and M2 macrophages were obtained after 48 h stimulation. RAW264.7 were plated at 10^6 in 6 cm culture dishes and stimulated with 20 ng/ml IL-4 for 48 h to differentiate into M2 macrophages.

### Ethics of clinical sample collection

This study has been approved by the Ethics Committee of Xinhua Hospital. Seven patients and the remaining 40 patients were diagnosed as ICC and underwent radical resection in Xinhua Hospital.

### In vitro co-culture and Transwell

The treated ICC cells were plated in a 6-well plate at a rate of 100w/well, and 3 replicate groups were set. The medium was replaced with serum-free RMPI-1640 medium for 48 h, and then the culture supernatant was collected as ICC cell conditioned medium (ICC-CM). After serum-free culture of induced M2 macrophages for 12 h, spread 50w/well in the upper chamber of Transwell (Corning, 8uM, 3422) covered or not covered with Matrigel (Beyotime, c0372) and set 3 experimental wells and 3 control wells for each cell line. When the cells adhered to the bottom, the lower chamber of the experimental well was replaced with a 1:1 mixture of ICC-CM and serum-free RMPI-1640 culture medium, and the lower chamber of the control well was replaced with serum-free RMPI-1640 culture medium. After 48 h of culture, the cells were fixed with paraformaldehyde and stained with crystal violet to observe the changes of migration and invasion ability. In studying the effects of LTB4 and 5-HETE on the migration and invasion ability of macrophages, the lower chamber was replaced with a specific concentration of LTB4 or 5-HETE in serum-free RPMI-1640 medium. The remaining steps were the same as above.

### Western blot

Whole cell proteins were extracted using RIPA buffer (Beyontime) with the addition of protease inhibitor cocktail and phosphatase inhibitors. Proteins were fractionated by SDS-PAGE and transferred to PVDF membranes. The membranes were incubated with 5% skim milk in TBST for 1 h. The primary antibodies and dilution times used in this study are as follows: ALOX5: Proteintech, 10,021–1-Ig, 1:1000; GAPDH: Proteintech, 10,494–1-AP, 1:2000; BLT1: ABclonal, 1:1000; BLT2: ABclonal, A15479, 1:1000; PIK3CA: ABclonal, A12484, 1:1000; Phospho-PI3K: ABclonal, AP0854, 1:1000; Phospho-AKT-T450: ABclonal, AP0980, 1:1000; Phospho-AKT-S473: ABclonal, AP0637, 1:1000; AKT: Cell Signaling Technology, C67E7, 1:1000; p-mTOR: Proteintech, 67,779–1-Ig, 1:2000; mTOR: Proteintech, 28,273–1-AP, 1:2000; E-cadherin: Servicebio, GB11868, 1:1000; N-cadherin: Proteintech, 22,018–1-AP, 1:2000; Snail: ABclonal, A5243, 1:1000; Vimentin: Proteintech, 10,366–1-AP, 1:2000. PVDF membranes were incubated overnight at 4 °C in the above antibodies. The membrane was washed three times for 10 min each and washed with 1:2000 diluted horseradish peroxidase conjugated anti-mouse or anti-rabbit antibody for 1 h. Blots were developed with an ECL system (Amersham Biosciences).

### ELISA

Cell conditioned medium LTB4 and 5-HETE was quantified using the LTB4 kit (Sangon Biotech) and the 5-HETE kit (Cusabio Biotech). The prepared cell conditioned medium was diluted to appropriate fold and added to a microplate coated with antibody against LTB4 and 5-HETE. Absorbance measurements were performed on LTB4 and 5-HETE using a microplate reader (SpectraMax).

### RNA extraction and RT-qPCR

Total RNA of cells or tissues was extracted using EZB RNA extraction kit. After reverse transcription into cDNA by reverse transcription reagent, the cDNA was added into qPCR system, and the signal value was detected by PCR instrument. Β-ACTIN was used as an internal reference. RNA primer sequences: ALOX5: F-ACAAGCCCTTCTACAACGACT

R-AGCTGGATCTCGCCCAGTT.

### In vivo xenograft tumor model

First, 5* 10^6 CCLP-1 cells suspended in 100uL PBS were injected subcutaneously into the flank of female BALB/c athymic nude mice (4–6 weeks old). When tumors reached a volume of 0.2-0.3 cm^3^, mice were divided into 4 groups (5 mice per group): Blank, Zileuton (Selleck), PLX3397 (Selleck), Zileuton + PLX3397. Zileuton: Zileuton: 200 mg/kg/day by oral gavage daily for 28 days; PLX3397: 50 mg/kg/2 days by oral gavage every other day for 14 days. After 4 weeks of continuous treatment, the tumor size and number of mice in each group were detected, and the abundance of ALOX5 and macrophages were observed by multiplex immunofluorescence technique and flow cytometry. Tumor volume was calculated using the following formula: Volume = (length * width^2^)/2.

### Flow cytometry

The tumor tissues were cut into 1.0mm3 tissue pieces and washed with PBS. At 37 °C, an appropriate amount of pancreatic enzyme digestive fluid was used to digest tissues for 20min. Digestion was terminated using DMEM with a double pancreatic enzyme volume, followed by filtration and collection of cells using a 70um filter. After centrifugation at 500g for 10min, the cell precipitate was re-suspended with 500uL buffer for flow cytometry analysis. The products used are as follows: Fixable viability dye (eF780): eBioscience, 65–0865-14; CD11b (PE/Cy7): eBioscience, 25–0112-82; F4/80 (FITC): BioLegend, 123,108; CD163: Proteintech, 16,646–1-AP; anti-rabbit Alexa Fluor 594: Thermofisher, A21207).

### Statistical analysis

Statistical analysis was performed using R4.1.3 software and GraphPad Prism software package (v.8.0). Results were expressed as mean plus or minus standard deviation, differences between two groups were analyzed using unpaired or paired Student's t-test. Differences between three or more groups were analyzed using one-way ANOVA. P < 0.05 was considered statistically significant.

## Results

### Single cell sequencing analysis reveals the distribution of macrophages in ICC

TAM infiltration in the tumor immune microenvironment was characterized by analysis of single-cell sequencing data (GSE138709) (5 ICC cancer tissues and 3 paracancerous tissues).

**Fig. 1:**
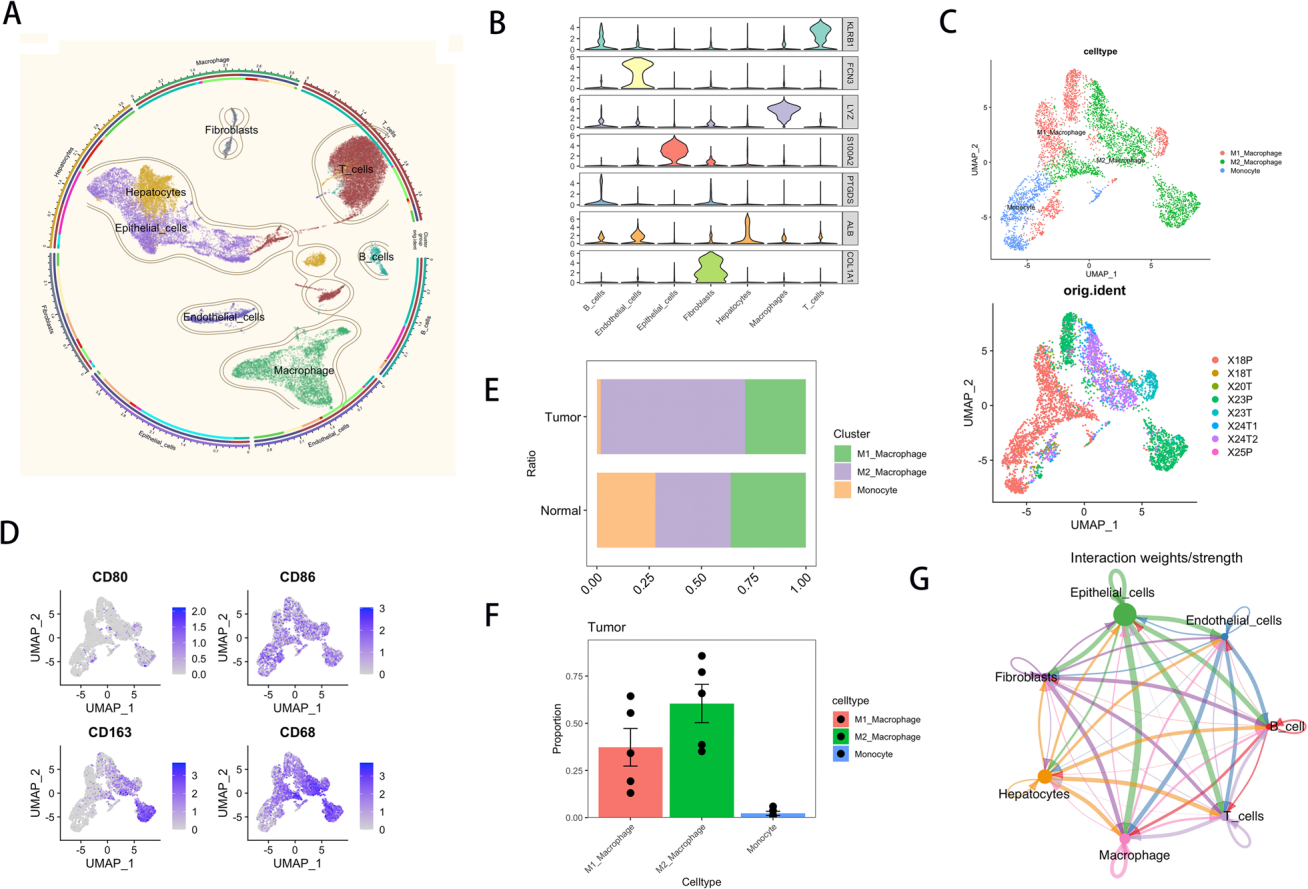
M2 macrophages mainly infiltrated in ICC tissues and interacted with epithelial cells. **a** UMAP plot showed that all cells are clustered into 7 cell types. **b** Violin plot shows the marker genes for each cell subset. **c** UMAP plot of macrophages extracted for the second reduction clustering. **d** UMAP plot showed the distribution and expression of macrophage marker genes in macrophage subsets. **e**, **f**) The proportional diagram of three cell types in tumor samples and normal samples. **g** Chord diagram showing the interactions between the various subpopulations analyzed by Cellchat package

First, all cells were clustered into 7 cell subpopulations, and the dimensionality reduction cluster plot is shown in Fig. 1a. Marker genes of 7 cell clusters were shown in Fig. 1b. Macrophage subpopulations were then extracted and re-clustered into M2 macrophages, Ml macrophages, and monocytes as shown in Fig. 1c-d. Analysis of the proportion of three types of cells in tumor samples showed that M2 macrophages were mainly infiltrated in tumor tissues, while M1 macrophages were mainly infiltrated in adjacent tissues, as shown in Fig. 1e, f. Tumor samples were extracted and the interaction between each cell subset was performed using the Cellchat package. The results show that the interaction axis with epithelial cells as ligand cells and macrophages as receptor cells is significantly enriched, as shown in Fig. 1g. In conclusion, we observed more infiltration of M2 macrophages in ICC tissues and a significant interaction between epithelial cells and TAM in cancer tissues.

We then verified the above findings in paired ICC tissues. We found that the density of CD68 in ICC tissues was significantly higher than that in normal tissues (Additional file 1: Fig. S1a). At the same time, the density and intensity of CD206 and CD163 in ICC tissues were higher than those in normal tissues, and they were obviously expressed in tumor stroma (Additional file 1: Fig. S1b, c). This means that M2 macrophages are more infiltrated in ICC tissue.

### Spatial distribution of epithelial cells and TAM in ICC samples.

**Fig.2:**
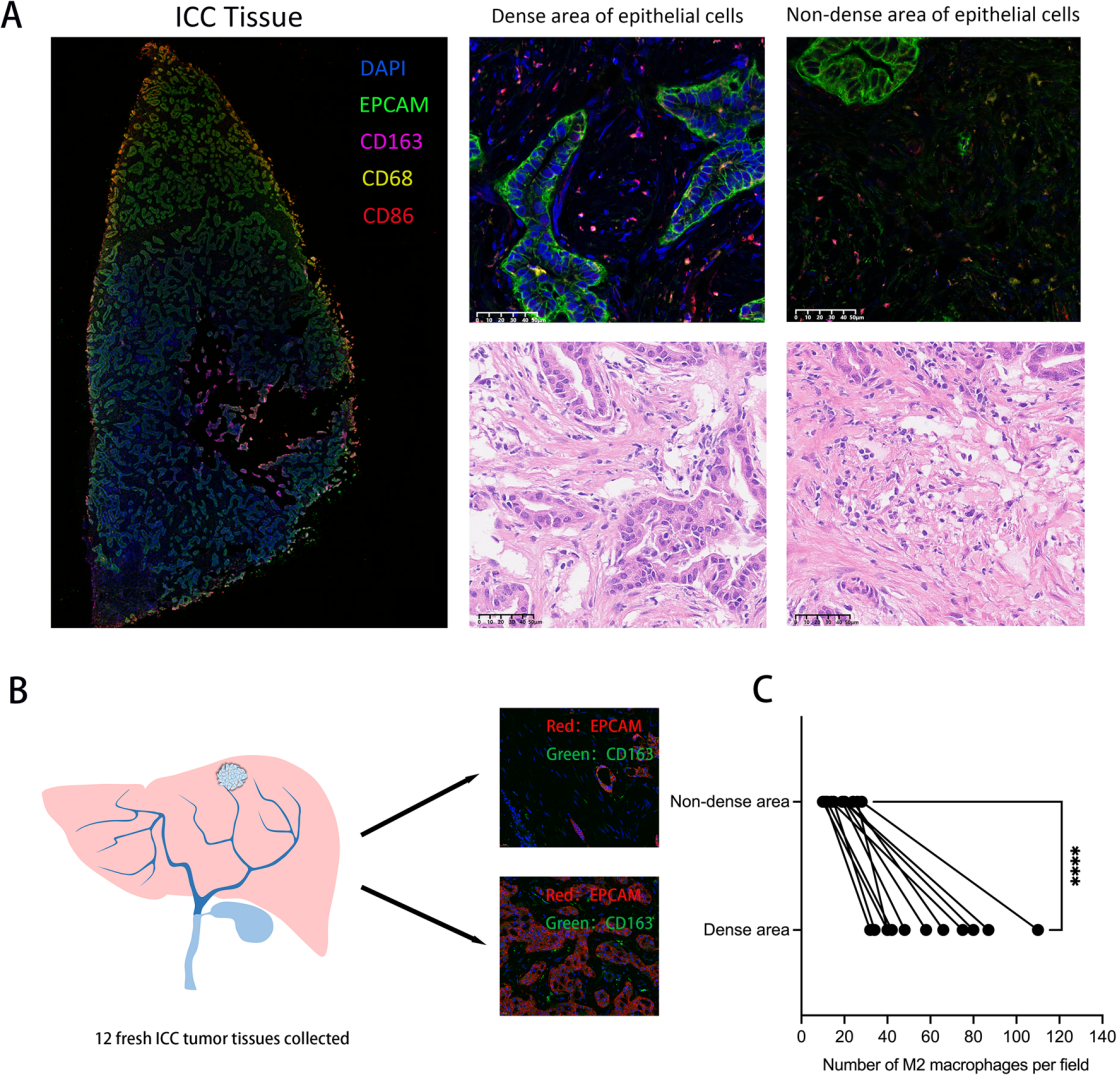
Association between spatial localization of epithelial cells and TAMs. **a** Multiplex immunofluorescence staining was used to analyze the spatial distribution of TAM in dense and sparse areas of epithelial cells. (Markers: EPCAM: epithelial cells; CD68: macrophages; CD86: M1 macrophages; CD163: M2 macrophages) **b** Model diagram of multiplex immunofluorescence staining analysis of 12 ICC tissues. **c** Paired diagram of the number of M2 macrophages in dense and non-dense epithelial areas. (*P < 0.05, **P < 0.01, ***P < 0.001, ****P < 0.0001)

The spatial distribution of epithelial cells and TAM in ICC was analyzed by multiple immunofluorescence staining. EPCAM is one of the most commonly used epithelial cell markers and CD68 is the most recognized macrophage surface marker. CD86 and CD163 are markers of M1 and M2 macrophages, respectively. Epithelial cells in ICC tissues were unevenly distributed, while macrophages infiltrated the stroma between epithelial cells. It was found that that infiltration of M2 macrophage was significantly higher in dense epithelial area than in sparse epithelial areas (Fig. 2a). Subsequently, we collected 12 fresh ICC tissues for multiple immunofluorescence analysis. Dense and non-dense areas (40x) of epithelial cells were selected for each sample (Fig. 2b, Additional file 2). The number of M2 macrophages in each region was counted and compared. The result showed that the abundance of M2 macrophages in the region with dense epithelial cells was significantly higher than that in the region with sparse epithelial cells (Fig. 2c). These results suggest that epithelial cells may recruit M2 macrophages and regulate the spatial localization of M2 macrophages.

### ALOX5 expression level correlates with TAM infiltration abundance.

**Fig. 3:**
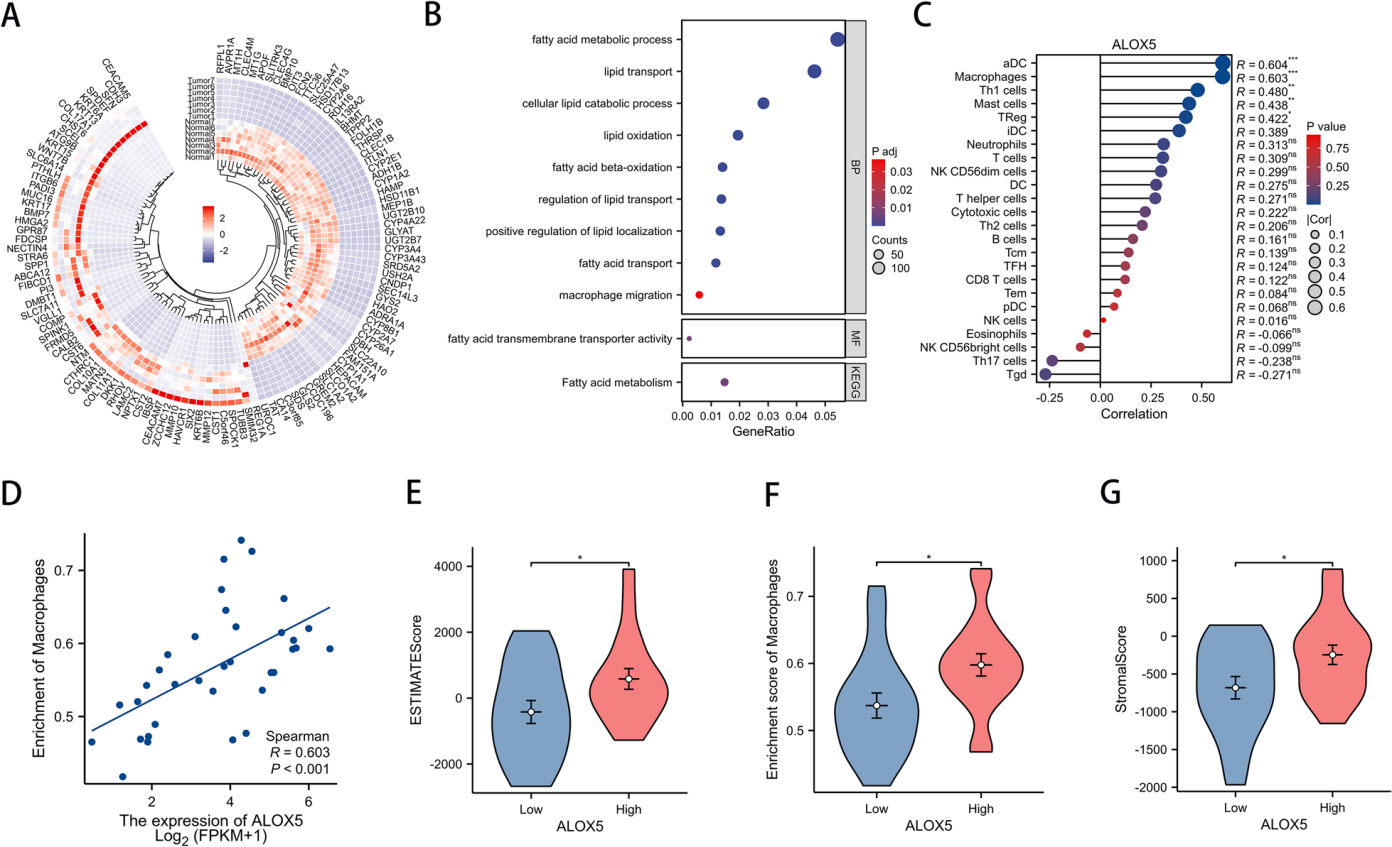
Bulk-RNA sequencing screened out lipid metabolism-related genes associated with TAM infiltration. (a) Differential expressed genes of 7 pairs of ICC tissues were displayed by heatmap. **b** Bubble diagram showing GO/KEGG enrichment analysis results. **c** Lollipop plots display the correlation of ALOX5 expression level with immune cells infiltration. **d** Scatter plot of correlation between ALOX5 expression level and macrophage infiltration abundance. **e**–**g**) Differences in macrophage infiltration scores between high and low ALOX5 expression groups were analyzed based on 3 different methods for evaluating immune infiltration. (*: P < 0.05, **: P < 0.01, ***: P < 0.001)

**Fig. 4:**
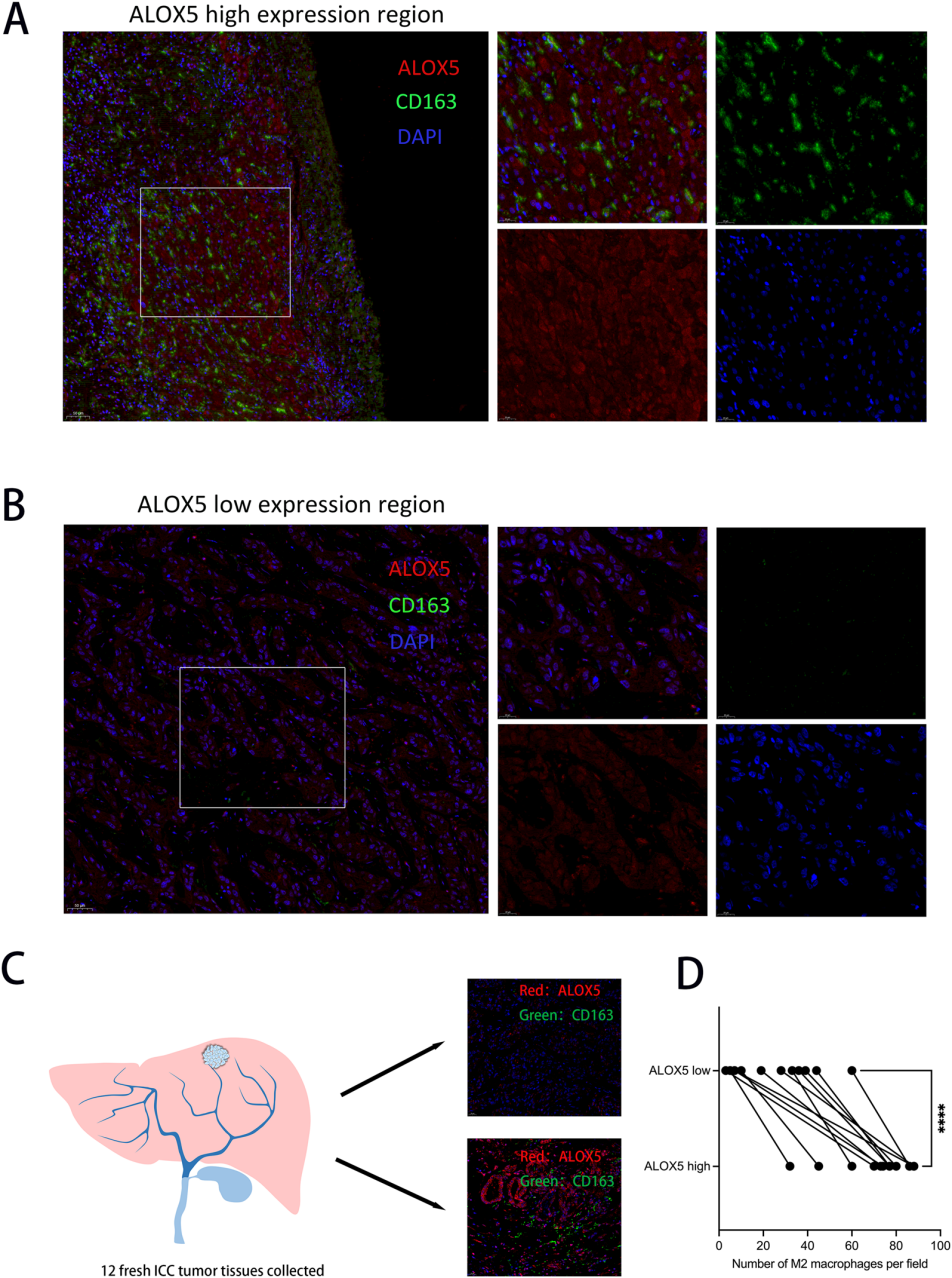
Expression level of ALOX5 was correlated with the abundance of M2 macrophage infiltration in ICC tissues. **a** Multiple immunofluorescence staining images demonstrating the abundance of M2 macrophage infiltration in regions with high ALOX5 expression. **b** Multiple immunofluorescence staining images demonstrating the abundance of M2 macrophage infiltration in regions with low ALOX5 expression. **c** Model diagram of multiplex immunofluorescence staining analysis of 12 ICC tissues. **d** Paired diagram of the number of M2 macrophages in ALOX5 high and low expression areas. (*P < 0.05, **: P < 0.01, ***: P < 0.001, ****: P < 0.0001)

The correlation between lipid metabolism related genes and immune cells infiltration was analyzed by bulk RNA data. The differentially expressed genes between 7 pairs of ICC and adjacent tissues were enriched by GO/KEGG analysis (Fig. 3a, b, Additional file 3). The results showed a significant enrichment of lipid metabolism and macrophage migration. Subsequently, CHOL tumor samples of TCGA database data were extracted, and all differential expressed lipid metabolism-related genes were subjected to immune cell infiltration correlation analysis. We found a significant positive correlation between ALOX5 expression and macrophage infiltration abundance (Fig. 3c–g). Multiple immunofluorescence analysis showed that TAM was more abundant in areas with high ALOX5 expression, while TAM infiltration was less in areas with low ALOX5 expression (Fig. 4a, b). After multiple immunofluorescence analysis of 12 fresh ICC tissues, high and low expression regions of ALOX5 were selected for each sample (Fig. 4c, Additional file 4). The result showed that the abundance of M2 macrophages in ALOX5 high expression region was significantly higher than that in ALOX5 low expression region (Fig. 4d). This suggests that ALOX5 may recruit M2 macrophages through some mechanism.

### ALOX5 expression level was up-regulated in ICC

**Fig. 5:**
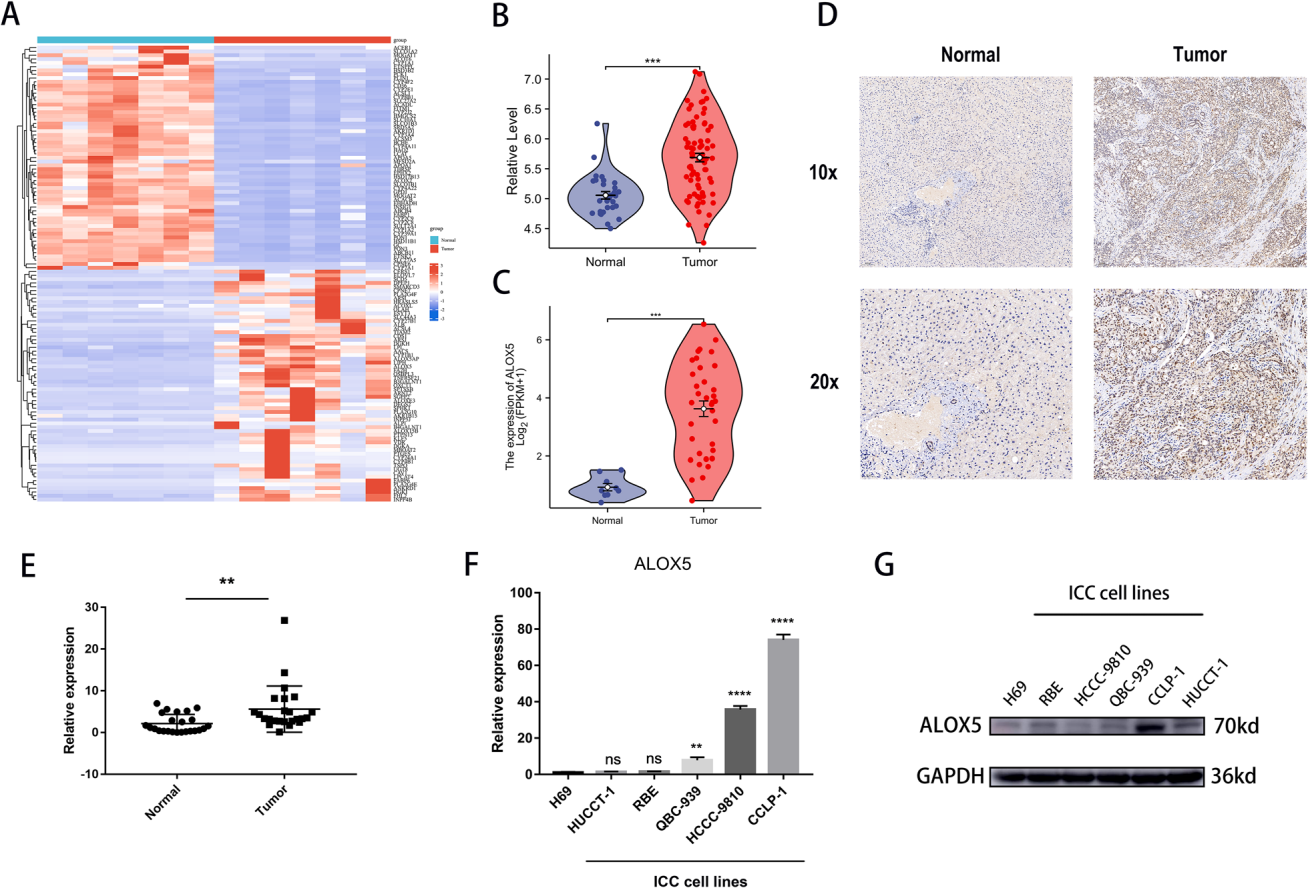
Evaluation of the expression level of ALOX5 in tissue samples and cell lines. **a** The heatmap showed the expression of 120 lipid metabolism-related genes with the most significant fold changes. **b**, **c** Violin plots showed significant upregulation of ALOX5 expression level in EMBI and TCGA databases. **d** Immunohistochemical image showing ALOX5 expression and localization in ICC and adjacent normal tissues. **e** Dot plot showing the relative expression levels of ALOX5 mRNA in 28 pairs of ICC patient tissues. **f** Histogram demonstrated ALOX5 RNA expression levels in 6 cell lines (H69: normal bile duct epithelial cell line; HUCCT1, RBE, QBC-939, HCCC-9810, CCLP-1: ICC cell lines). **g** WB showed the protein expression levels of ALOX5 in 6 cell lines. (*: P < 0.05, **: P < 0.01, ***: P < 0.001, ****: P < 0.0001)

The expression level of ALOX5 in ICC was determined by high-throughput sequencing and detection of clinical samples and cell lines. ALOX5 expression level was significantly upregulated in the sequencing result of 7 pairs of ICC tissues (Fig. 5a). Its expression level was also significantly up-regulated in EMBI and TCGA database (Fig. 5b, c). Immunohistochemical analysis showed that ALOX5 was significantly upregulated in ICC tissues and expressed mainly in the cytoplasm. In contrast, ALOX5 was almost absent in normal bile duct tissues (Fig. 5d). RT-qPCR confirmed that its mRNA expression level was significantly up-regulated in 28 pairs of ICC samples (Fig. 5e). ALOX5 mRNA and protein levels were significantly upregulated in ICC cell lines compared with normal bile duct epithelial cell line H69 (Fig. 5f–g).

### ALOX5 recruits M2 macrophages in vitro.

**Fig. 6:**
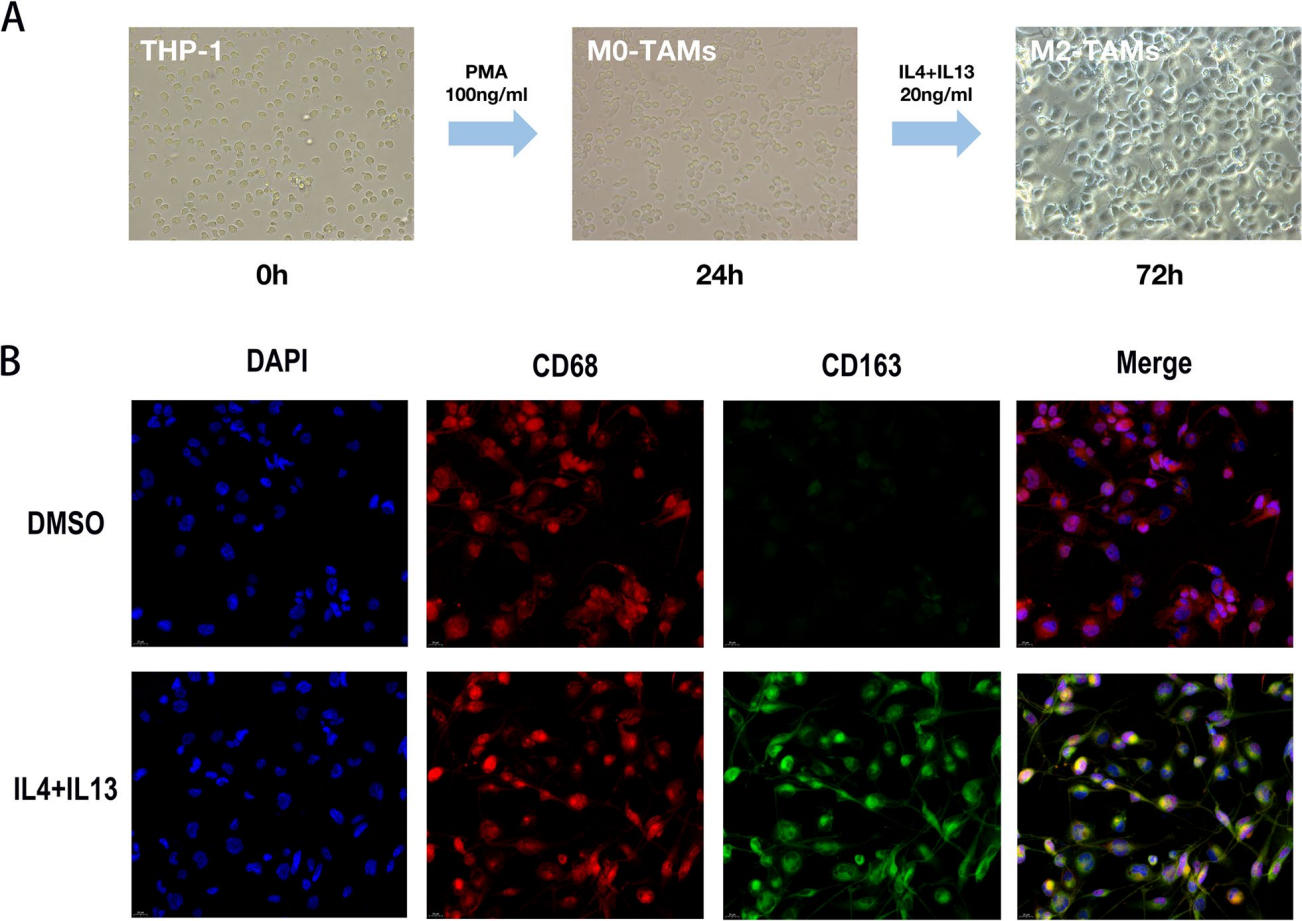
M2 macrophage induction in vitro. **a** Conceptual map of M2 macrophage induction. **b** Immunofluorescence images demonstrated the induction efficiency of M2 macrophages

**Fig. 7:**
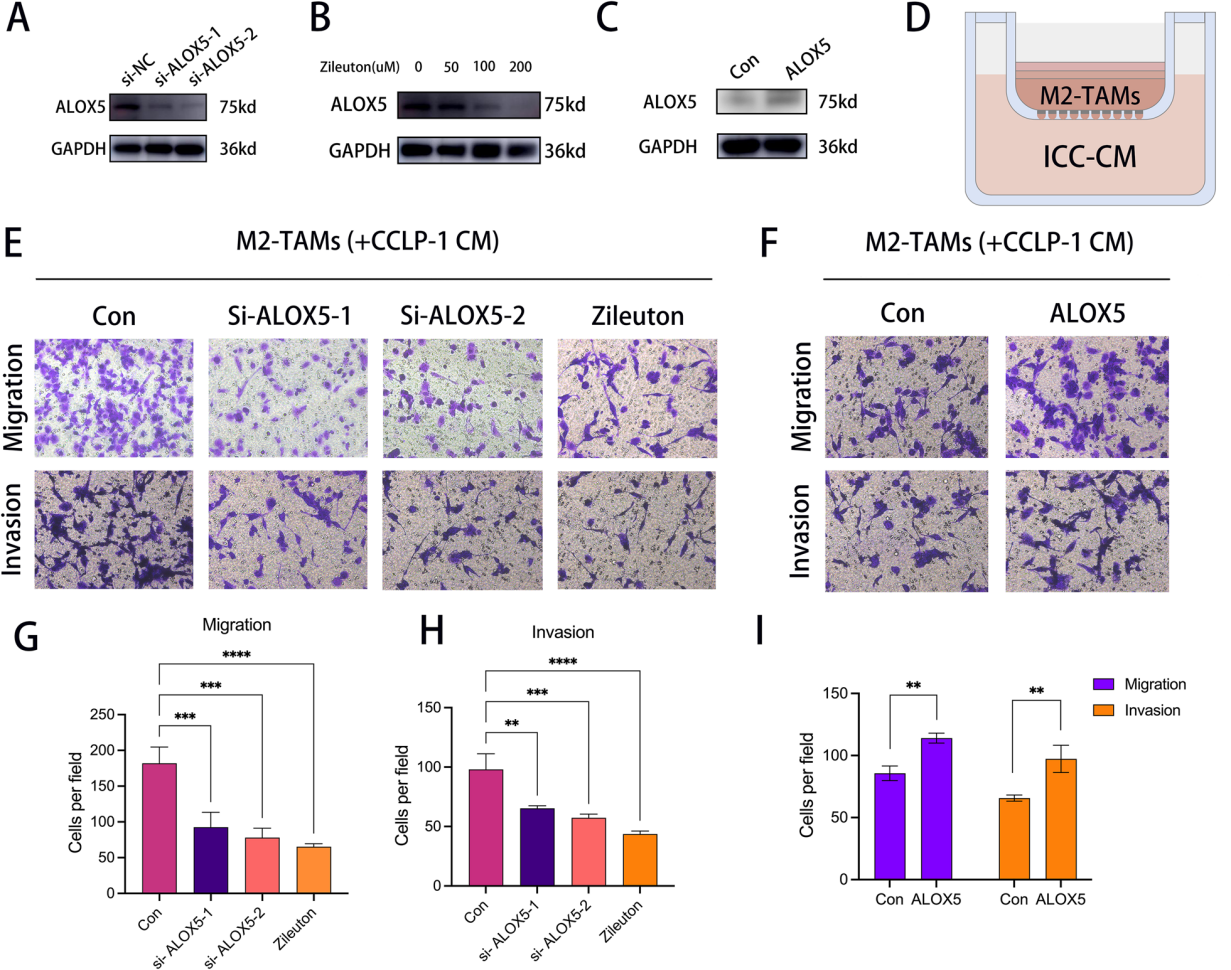
In vitro co-culture experiments revealed that ALOX5 recruited M2 macrophages. **a** WB showed the change of protein expression level after transfection of si-ALOX5. **b** WB showed the change of protein expression level after treatment of CCLP-1 cells with Zileuton. **c** WB demonstrated the change of protein expression level after transfection of ALOX5 overexpression plasmid in HUCCT-1 cells. **d** Transwell pattern diagram. **e**, **f** Transwell image displayed of the effect of conditioned medium on the migration and invasion of M2 macrophages. **g**, **h** Bar graph showing the effect of ICC-CM on M2 macrophage migration and invasion capacity after knockdown of ALOX5 or Zileuton treatment. **i** Bar graph showing the effect of ICC-CM on M2 macrophage migration capacity after overexpression of ALOX5. (*: P < 0.05, **: P < 0.01, ***: P < 0.001, ****: P < 0.0001)

First, we induced THP-1 cells to become adherent M2 macrophages by adding PMA, IL-4 and IL-13 to THP-1 cells. The induction efficiency was detected by immunofluorescence staining (Fig. 6a, b). The knockdown efficiency of si-ALOX5 was detected at protein level after transfection into CCLP-1 cells (Fig. 7a). ALOX5 protein level was detected after addition of ALOX5 inhibitor Zileuton to CCLP-1 cells (Fig. 7b). After transfection of the ALOX5 overexpression plasmid, the overexpression efficiency was examined at the protein level (Fig. 7c). The in vitro co-culture pattern is shown in Fig. 7d. The conditioned medium (CM) of CCLP-1 cells after treatment and the CM of control group were collected to detect their effect on the migration and invasion ability of M2 macrophages (Fig. 7e, f). The recruitment of M2 macrophages by ICC-CM after knockdown of ALOX5 or inhibitor treatment was significantly reduced, while the recruitment of ICC-CM after overexpression of ALOX5 was significantly enhanced (Fig. 7g–i).

### ALOX5 downstream metabolite LTB4 promotes migration and invasion of M2 macrophages.

**Fig. 8:**
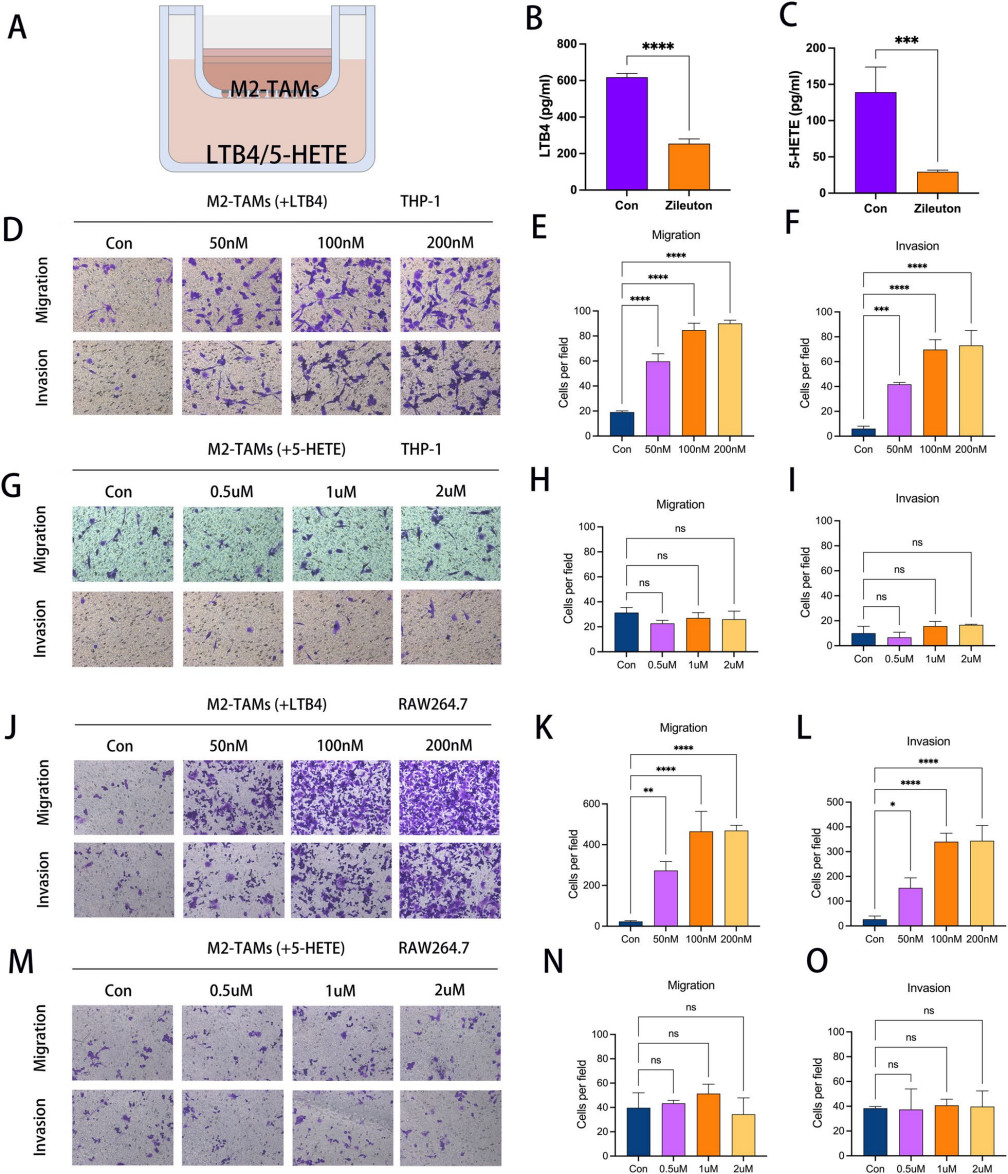
LTB4, but not 5-HETE, promoted migration and invasion of M2 macrophages. **a** Transwell pattern diagram. **b** Histogram showed the concentration of LTB4 in ICC-CM at an appropriate dilution. **c** Histogram showed the concentration of 5-HETE in ICC-CM at an appropriate dilution. **d** Transwell was used to detect the effect of different concentration gradients of LTB4 on the migration and invasion ability of M2 macrophages derived from THP-1. **e** Histogram showing the effect of different concentrations of LTB4 on the migration capacity of M2 macrophages derived from THP-1. **f** Histogram showing the effect of different concentrations of LTB4 on the invasion capacity of M2 macrophages derived from THP-1. **g** Transwell images showing the effect of different concentration gradients of 5-HETE on the migration and invasion ability of M2 macrophages derived from THP-1. **h** Histogram showing the effect of different concentrations of 5-HETE on the migration capacity of M2 macrophages derived from THP-1. **i** Histogram showing the effect of different concentrations of 5-HETE on the invasion capacity of M2 macrophages derived from THP-1. **j** Transwell was used to detect the effect of different concentration gradients of LTB4 on the migration and invasion ability of M2 macrophages derived from RAW264.7. **k** Histogram showing the effect of different concentrations of LTB4 on the migration capacity of M2 macrophages derived from RAW264.7. **l** Histogram showing the effect of different concentrations of LTB4 on the invasion capacity of M2 macrophages derived from RAW264.7. **m** Transwell images showing the effect of different concentration gradients of 5-HETE on the migration and invasion ability of M2 macrophages derived from RAW264.7. **n** Histogram showing the effect of different concentrations of 5-HETE on the migration capacity of M2 macrophages derived from RAW264.7. **o** Histogram showing the effect of different concentrations of 5-HETE on the invasion capacity of M2 macrophages derived from RAW264.7. (*P < 0.05, **P < 0.01, ***P < 0.001, ****P < 0.0001)

Next, we examined the effects of ALOX5 downstream metabolites, LTB4 and 5-HETE, on the migration and invasion of M2 macrophages (Fig. 8a). First, we examined the approximate levels of LTB4 and 5-HETE in CCLP-1 conditioned medium after the addition of the inhibitor Zileuton (Fig. 8b, c). Subsequently, we added different concentration gradients of LTB4 and 5-HETE into the lower chamber of transwell to detect their effects on the migration and invasion ability of M2 macrophages (derived from THP-1). The results showed that LTB4 significantly up-regulated the migration and invasion ability of M2 macrophages (Fig. 8d–f), while 5-HETE did not (Fig. 8g–i). Next, we performed the same experiment on M2 macrophages derived from RAW264.7. The results were consistent with the previous ones. LTB4 instead of 5-HETE is the major downstream metabolite of the recruitment of M2 macrophages (Fig. 8j–o).

### LTB4 promotes M2 macrophage migration and invasion through PI3K-AKT pathway

**Fig. 9:**
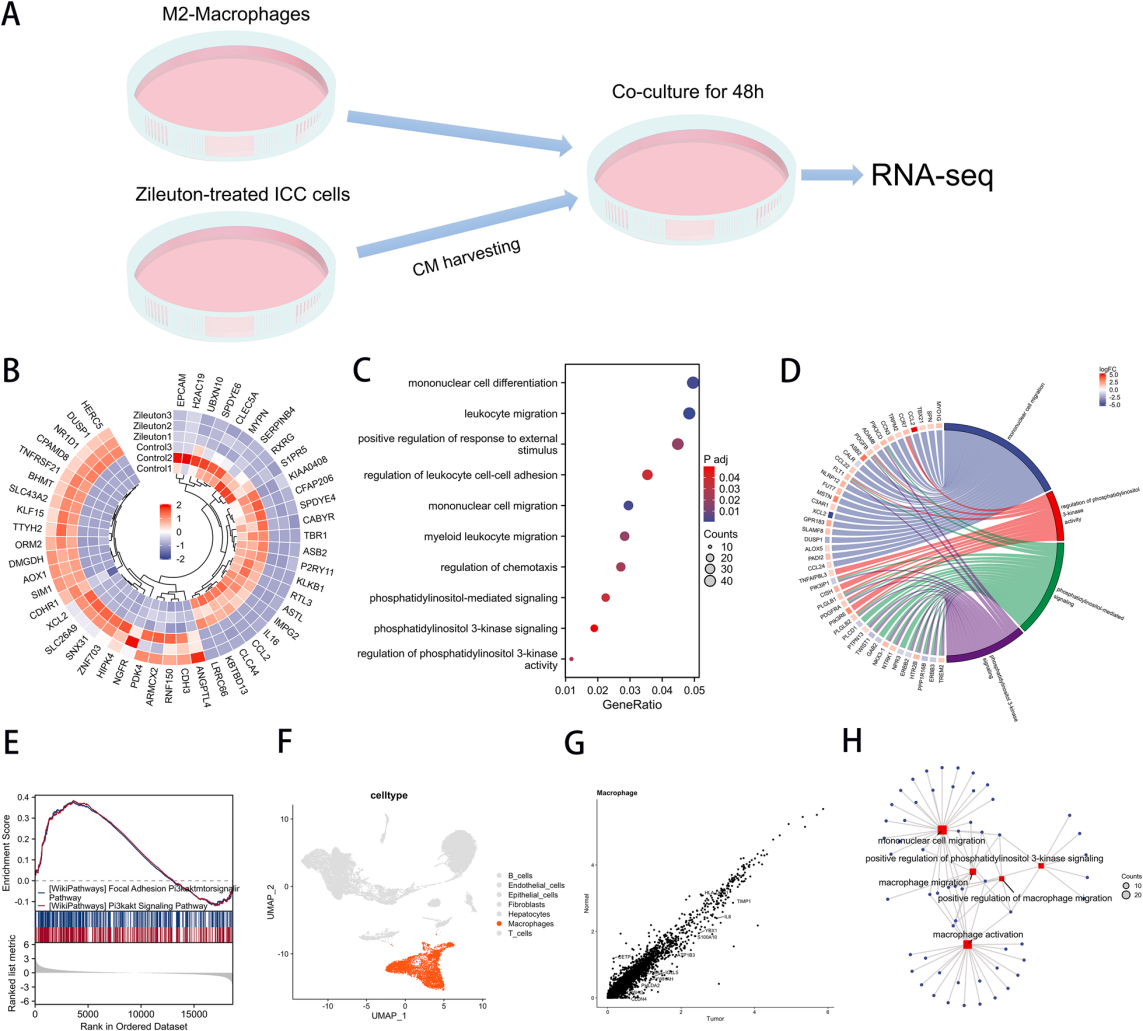
Bulk-RNA sequencing combined with single-cell analysis revealed key downstream pathways that enhanced migration and invasion of M2 macrophages. **a** In vitro co-culture pattern diagram. **b** Heat map showing differential expressed gene expression of M2 macrophages after secondary sequencing in Zileuton-CM or Con-CM co-culture. **c** GO/KEGG analysis shown by bubble plot. **d** Chord plot of pathway-specific GO/KEGG analysis. **e** GSEA analysis between high and low ALOX5 expression groups in TCGA-CHOL dataset. **f** Macrophages were highlighted in UMAP plot. **g** Volcano plot showed differentially expressed genes between tumor and normal samples in macrophage subsets. **h** Network diagram showing GO/KEGG analysis of differentially expressed genes in macrophage subsets

Subsequently, we construct an in vitro co-culture model (Fig. 9a). The specific mechanism by which ALOX5 affects macrophage migration was examined by RNA sequencing. Sequencing results showed that compared with Zileuton-CM group, Con-CM group was significantly enriched in macrophage migration, PI3K pathway (Fig. 9b, c, Additional files 5, 6). Subsequently, we extracted tumor samples of TCGA-CHOL and divided them into high expression group and low expression group according to median ALOX5 expression value. Then the enrichment analysis was performed after differential analysis to verify the enrichment of PI3K pathway. GSEA analysis suggested a significant enrichment of the PI3K pathway (Fig. 9d). Macrophage subsets were extracted by single cell data analysis, and the difference between tumor group and normal group was analyzed. The results of enrichment analysis of differential expressed genes suggest that the PI3K pathway plays an important role in macrophage migration (Fig. 9e–h).

**Fig. 10:**
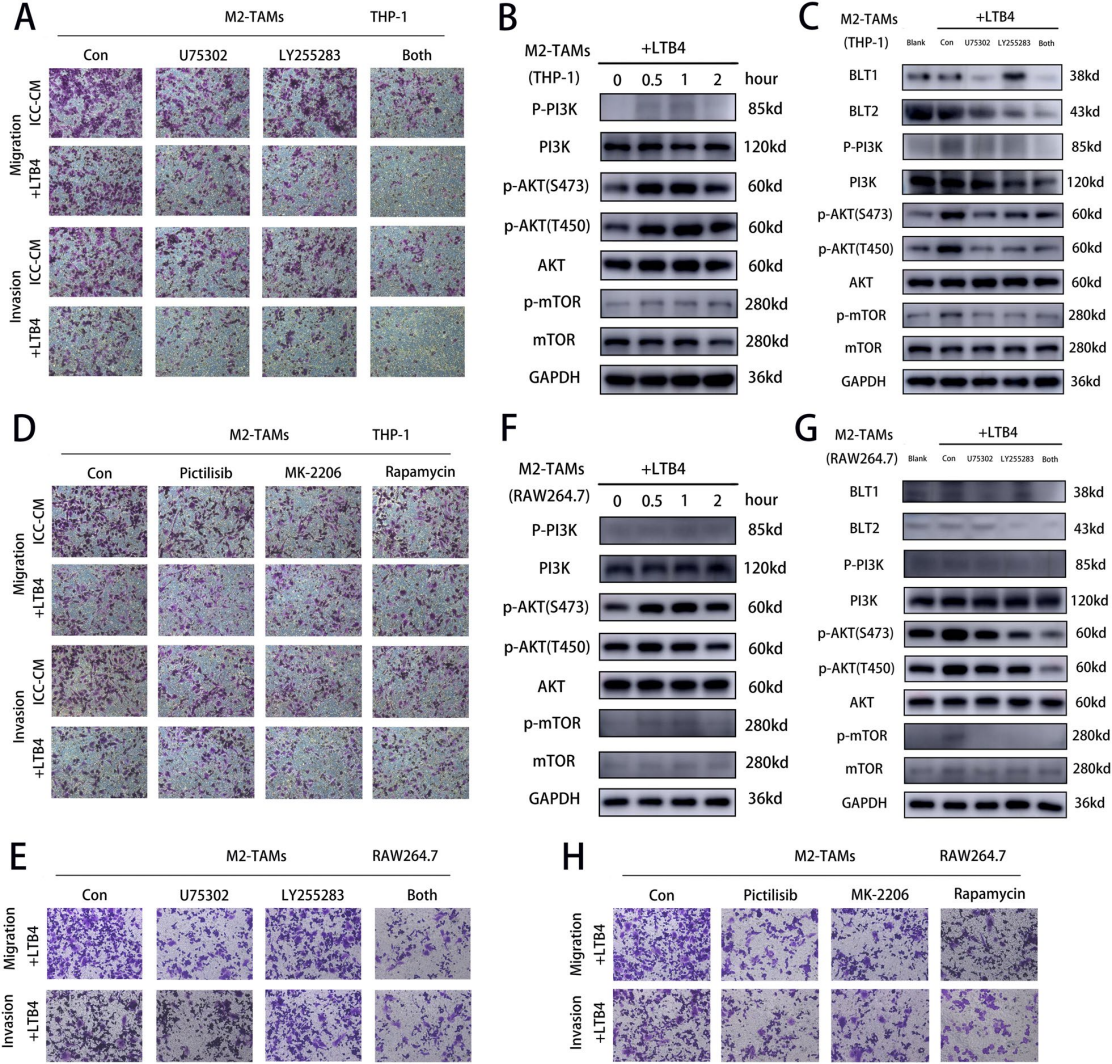
LTB4 activates PI3K pathway through BLT1/BLT2 to promote migration and invasion of M2 macrophages. **a** Transwell images showing the effect of addition of LTB4 on M2 macrophage migration and invasion in the presence of BLT1 or BLT2 inhibitors (derived from THP-1). **b** WB images demonstrating the expression of PI3K pathway proteins at different time after the addition of LTB4 to M2 macrophages derived from THP-1. **c** WB images showing expression of PI3K pathway proteins after addition of BLT1 and BLT2 inhibitors after addition of LTB4 to M2 macrophages derived from THP-1. **d** Transwell images demonstrating the effect of LTB4 addition on the migration and invasion of M2 macrophages (derived from THP-1) in the presence of PI3K, AKT, mTOR inhibitors. **e** Transwell images showing the effect of addition of LTB4 on M2 macrophage migration and invasion in the presence of BLT1 or BLT2 inhibitors (derived from RAW264.7). **f** WB images demonstrating the expression of PI3K pathway proteins at different time after the addition of LTB4 to M2 macrophages derived from RAW264.7. **g** WB images showing expression of PI3K pathway proteins after addition of BLT1 and BLT2 inhibitors after addition of LTB4 to M2 macrophages derived from RAW264.7. **h** Transwell images demonstrating the effect of LTB4 addition on the migration and invasion of M2 macrophages (derived from RAW264.7) in the presence of PI3K, AKT, mTOR inhibitors

Next, we carried out experimental verification. We first identified the role of LTB4R and LTB4R2 as the major receptors for LTB4 in the recruitment of M2 macrophages by LTB4. We first examined the effects of ICC-CM or LTB4 on the migration and invasion of M2 macrophages (derived from THP-1) treated with U75302 (LTB4R inhibitor) and LY255283 (LTB4R2) inhibitors. The results showed that the addition of either inhibitor could attenuate the recruitment of M2 macrophages by ICC-CM or LTB4 (Fig. 10a). Addition of both inhibitors significantly attenuated the recruitment of M2 macrophages by ICC-CM or LTB4. Next, we examined the changes in PI3K pathway proteins at different times after the addition of LTB4. The results showed that the PI3K pathway was activated. LTB4 treatment increased phosphorylation of PI3K, AKT and mTOR, the key effectors of PI3K signaling that are important for cell migration (Fig. 10b). Subsequently, we examined the activation of PI3K pathway proteins after the addition of LTB4R and LTB4R2 inhibitors. The results showed that inhibition of LTB4R and LTB4R2 significantly attenuated the activation of PI3K pathway (Fig. 10c). Finally, we examined the effects of three inhibitors of the PI3K pathway (PI3K inhibitor Pictilisib, AKT inhibitor MK2206, and mTOR inhibitor Rapamycin) on the ability of LTB4 to recruit M2 macrophages. The recruitment of M2 macrophages by LTB4 was significantly attenuated by inhibition of the PI3K pathway (Fig. 10d). We did the same experiments on M2 macrophages derived from RAW264.7 and the results were consistent with the above (Fig. 10e–h). This suggests that LTB4 recruits M2 macrophages via BLT1/BLT2/PI3K/AKT axis.

### In vivo efficacy study of targeting tumor-associated macrophages in combination with targeting ALOX5.

**Fig. 11:**
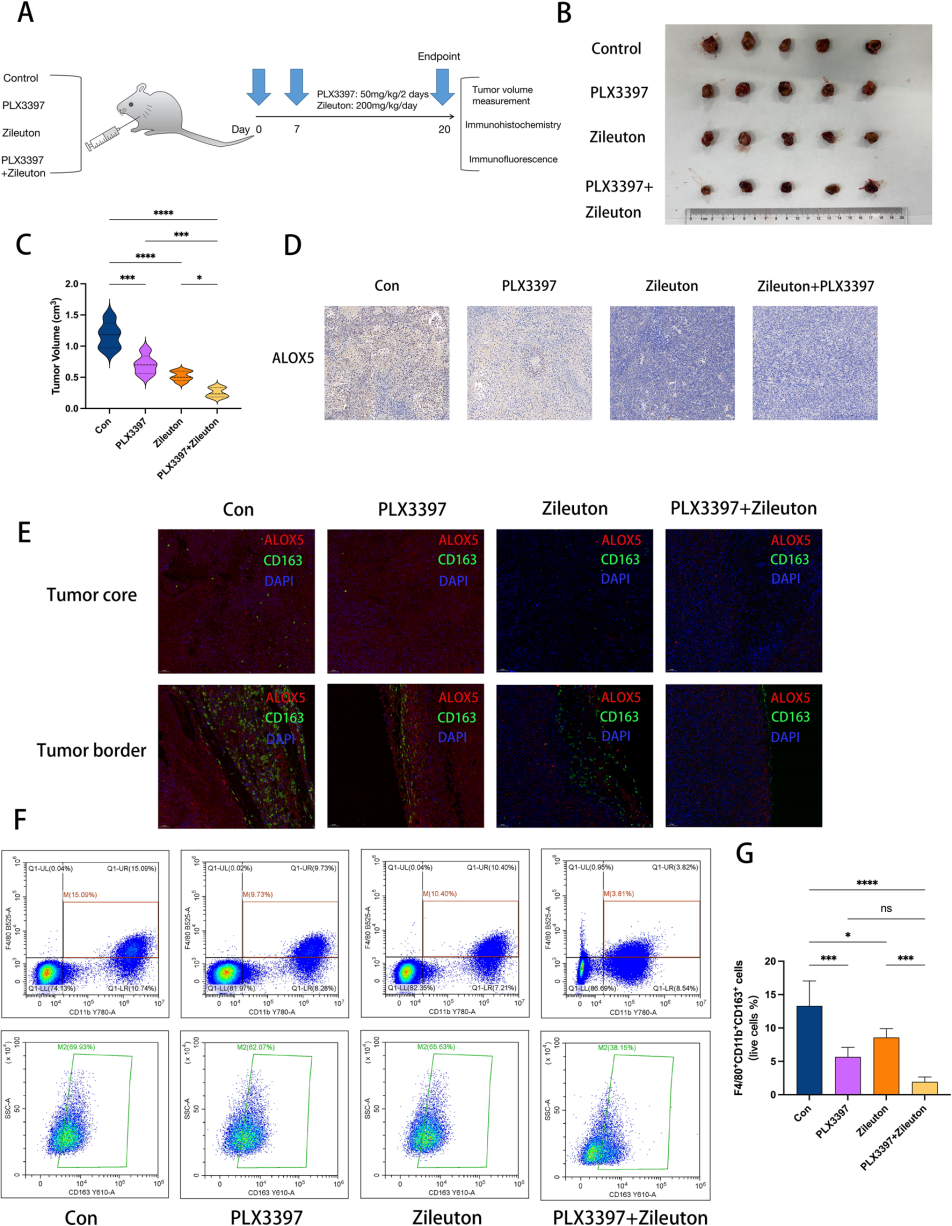
In vivo translational efficacy of Zileuton combined with PLX3397. **a** Pattern diagram. **b** Tumor images showing tumor size in each group. **c** Violin diagram demonstrated the change of tumor volume in different treatment groups. **d** Immunohistochemical images showing ALOX5 protein expression levels in each group. **e** Immunofluorescence staining images showing the intensity and density of ALOX5 and CD163 in each group. **f** Gating strategy of M2 macrophages in tumor tissues of four groups (Markers: CD11b, F4/80: macrophages; CD163: M2 macrophages) **g** Bar chart showing the infiltration ratio of M2 macrophages in tumor tissues of the four groups (CD11b + F4/80 + CD163 + cells/ living cells). (*: P < 0.05, **: P < 0.01, ***: P < 0.001, ****: P < 0.0001)

Subsequently, we explored the in vivo efficacy of a CSF1R inhibitor (PLX3397) in combination with an ALOX5 inhibitor (Zileuton). PLX3397 can block CSF1/CSF1R signaling, thereby inhibiting proliferation, differentiation and chemotaxis of TAMs. We implanted ICC cells subcutaneously into nude mice to test whether TAM inhibition could synergize with targeting ALOX5. Mice were randomly assigned to 4 treatment groups: blank control, Zileuton, PLX3397, Zileuton + PLX3397. Zileuton: 200 mg/kg/day by oral gavage daily for 14 days for a total of 672 mg; PLX3397: 50 mg/kg/2 days every other day for 14 days for a total of 42 mg (Fig. 11a). After 2 weeks of treatment, we found that the tumor volume in the combination group was significantly smaller than that in the monotherapy group and the control group. We believe that TAM inhibition enhances the killing response of Zileuton to ICC (Fig. 11b, c). Next, we detected the expression of ALOX5 by immunohistochemistry. The results showed a significant decrease in ALOX5 protein expression level in the Zileuton group and the combination group (Fig. 11d). By immunofluorescence, we found that the intensity of ALOX5 and CD163 was significantly reduced in the combination group, and the density of CD163-positive cells was significantly lower than that in the monotherapy group and the control group (Fig. 11e). Interestingly, the trend of CD163 + macrophages was consistent in both the central and marginal regions of the tumor. Subsequently, we performed flow cytometric analysis of each tumor tissue. As shown in Fig. 11f, CD11b + F4/80 + CD163 + cells were selected from living cells and their proportion in living cells was calculated. The results showed that the proportion of M2 macrophages decreased significantly in the Zileuton treatment group and the PLX3397 treatment group. The proportion of M2 macrophage infiltration in the combined treatment group was significantly decreased compared with the Zileuton treatment group and the control group, and showed a downward trend compared with the PLX3397 treatment group, but the difference was not significant (Fig. 11g). These results suggest that inhibition of TAM combined with targeting ALOX5 has clinical translational significance.

### Recruited M2 macrophages promote ICC progression

**Fig. 12:**
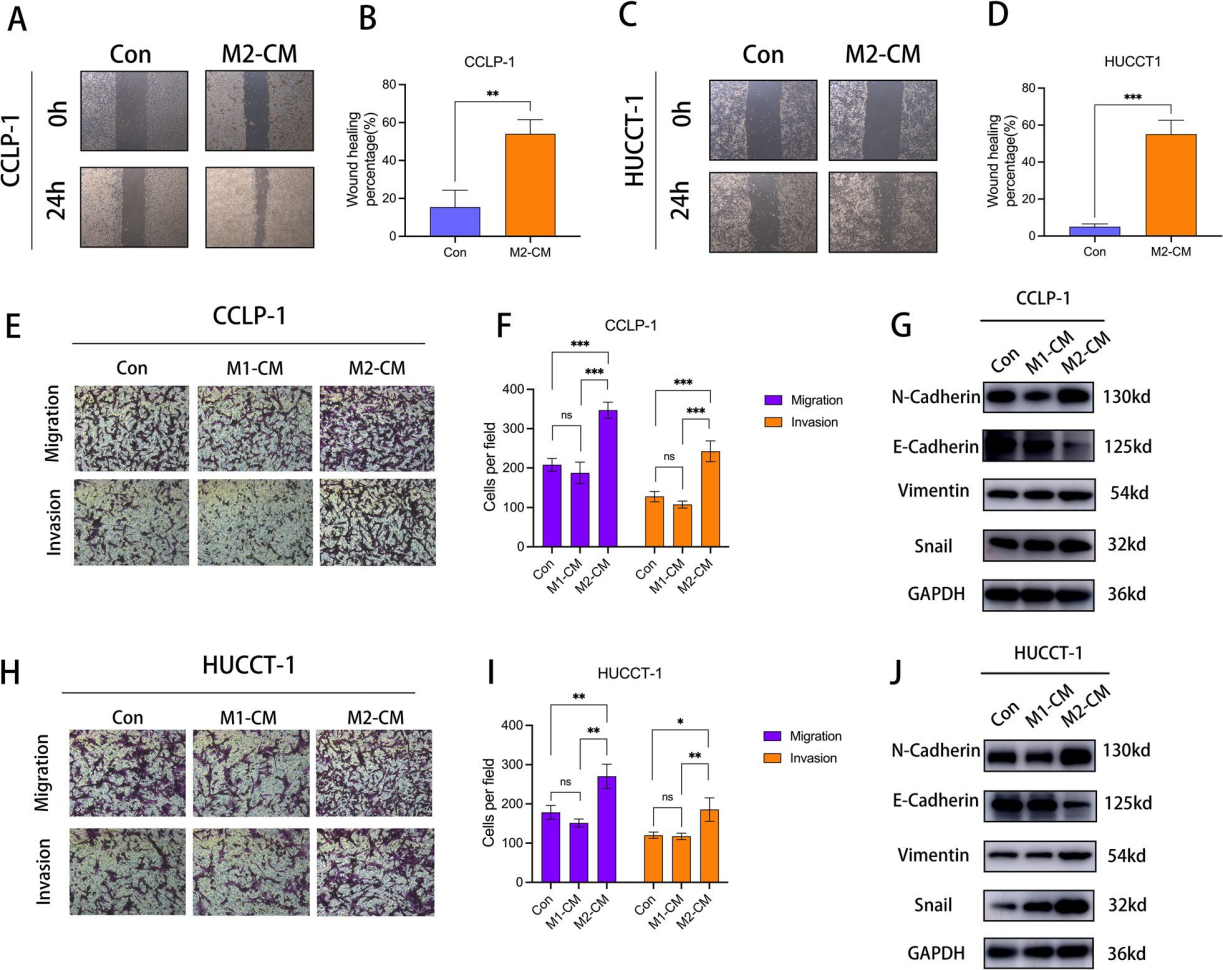
Infiltrating M2 macrophages promote ICC migration and invasion. **a** Wound healing test showed the effects of control, M0 and M2 macrophages on the migration ability of CCLP-1 cells. **b** Bar graph demonstrating wound healing rate for control and M2-CM treated CCLP-1 cells. **c** Wound healing test showed the effects of control, M0 and M2 macrophages on the migration ability of HUCCT-1 cells. **d** Bar graph showing wound healing rate for control and M2-CM treated HUCCT-1 cells. (e) Transwell images showing the effects of control, M0, and M2 macrophages on migration and invasion of CCLP-1 cells. **f** Histograms demonstrated the changes of migration and invasion ability of CCLP-1 cells in control group, M1-CM group and M2-CM group. **g** WB images showed the changes of EMT-related protein expression level in CCLP-1 cells after co-culture with control, M0 and M2 macrophages. **h** Transwell images showing the effects of control, M0, and M2 macrophages on migration and invasion of HUCCT-1 cells. **i** Histograms demonstrated the changes of migration and invasion ability of HUCCT-1 cells in control group, M1-CM group and M2-CM group. **j** WB images showed the changes of EMT-related protein expression level in HUCCT-1 cells after co-culture with control, M1 and M2 macrophages. (*: P < 0.05, **: P < 0.01, ***: P < 0.001, ****: P < 0.0001)

In order to verify the role of M2 macrophages in promoting the malignant progression of ICC cells, we established a co-culture model in vitro and examined the effects of M0 and M2 macrophages on the migration of ICC cells. Wound healing test showed that M2 macrophages significantly promoted ICC cell migration, while M0 macrophages had no similar effect (Fig. 12a–d). The results of transwell experiment are consistent with those of wound healing test, in either CCLP-1 (Fig. 12e, f) or HUCCT-1 cell line (Fig. 12h, i). Then we examined the expression level of EMT-associated proteins. The results showed that M2 macrophages promoted EMT of ICC cells in both CCLP-1 (Fig. 12g) and HUCCT-1 cell line (Fig. 12j).

## Discussion

Intrahepatic cholangiocarcinoma (ICC) is a primary liver cancer that occurs within the liver, between the bile duct and the secondary bile duct, and its incidence is increasing worldwide [25, 26]. Because it has the characteristics of both liver and biliary tract tumors, it is extremely invasive [27, 28]. The mechanism of ICC metabolic reprogramming regulating immune microenvironment is rarely studied, and it is novel to analyze the mechanism from the perspective of tumor metabolic reprogramming regulating immune microenvironment [29–32]. The combination of single cell and bulk sequencing is of great significance to elucidate the regulatory mechanism of immune microenvironment [33–35].

**Fig. 13:**
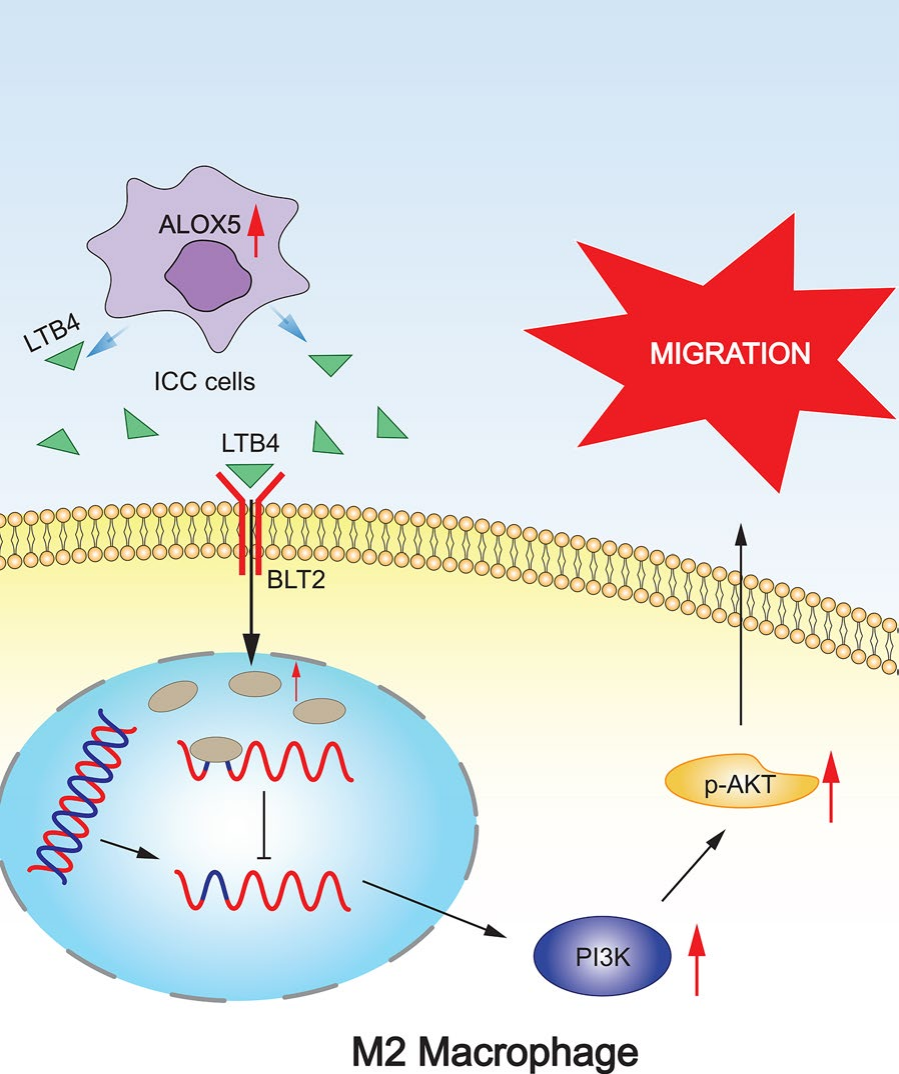
Schematic diagram of this study. Upregulated ALOX5 in ICC cells promotes M2-TAM infiltration through LTB4/BLT1/2/PI3K/AKT axis. LTB4 enters TME from ICC cells, binds to M2 macrophage surface receptor BLT1/BLT2, activates PI3K pathway and recruits M2 macrophages

In this study, we systematically investigated the mechanism of ALOX5/LTB4/TAM axis regulating ICC progression in the development of ICC by elucidating the new mechanism of ICC lipid metabolism reprogramming remodeling immune microenvironment (Fig. 13). First, we analyzed single-cell sequencing data to reveal ICC macrophage distribution patterns. Compared with normal tissues, M2 macrophages were predominant in tumor tissues in ICC immune microenvironment. Moreover, there was significant interaction between epithelial cells and macrophages. In spatial distribution, the infiltration of M2 macrophages in dense epithelial areas was significantly higher than that in sparse epithelial areas. Next, we verified that ALOX5 expression was significantly associated with M2 macrophage infiltration in ICC specimens by bulk sequencing analysis and multiplex immunofluorescence staining. After verifying that ALOX5 is highly expressed in ICC epithelial cells, we constructed an in vitro co-culture model. Co-culture experiments confirmed that LTB4, the downstream metabolite of ALOX5, promoted migration and invasion of M2 macrophages. Subsequently, we identified the key pathway responsible for the up-regulation of migration ability of M2 macrophages by sequencing after co-culture, combined with single-cell analysis: PI3K-AKT pathway. In vitro co-culture experiments confirmed that LTB4 promoted the migration ability of macrophages by binding to BLT1/BLT2 and activating PI3K-AKT pathway. Finally, we investigated the in vivo translational efficacy of a CSF1R inhibitor (PLX3397) in combination with an ALOX5 inhibitor (Zileuton). PLX3397, a commonly used CSF1/CSF1R signaling blocker, inhibits TAMs proliferation, differentiation, and chemotaxis. TAM inhibition enhances the killing response of Zileuton to ICC, and inhibition of TAM combined with targeting ALOX5 has clinical translational significance.

5-LOX expression and metabolites have been found to be upregulated in a number of cancers and have been shown to be associated with tumorigenesis, progression or drug resistance, including prostate, renal, breast, colorectal and pancreatic tumors. Pharmacological inhibition of the 5-LO pathway has an inhibitory effect on cancer cell proliferation [12, 36, 37]. This study demonstrates that ALOX5 is significantly upregulated in ICC epithelial cells and is barely expressed in normal bile ducts. This may be related to the specific pathogenesis of ICC. Inflammation is often an important factor in ICC pathogenesis [38]. Khophai demonstrated that inhibition of ALOX5 blocks the oncogenic pathway in CCA cells [39]. In addition to activating oncogenic signaling pathways in tumor cells, its role in TME cannot be underestimated due to the mediating properties of its metabolites. For example, studies have shown increased immune matrix expression of 5-LO and other distal enzymes involved in the synthesis of 5-LO-derived leukotrienes in human esophageal adenocarcinoma. This suggests that 5-LO signaling has a specific role in TME during tumor development and progression [40]. TAMs, as one of the most infiltrating immune cells, are involved in the progression of many malignancies, including ICC. Co-culture with M2-TAM enhances the EMT capacity of ICC cells, enhancing cell invasion and metastasis through AKT3/PRAS40 phosphorylation [41]. TAMs have immunosuppressive and chemotherapy-resistant effects that exacerbate tumor progression [42, 43]. The current research focuses on how TAM regulates the malignant phenotype of ICC cells, but how ICC cells interact with TAM to form TME that promotes tumor growth is still lacking. Our study fills this gap by demonstrating that upregulated ALOX5 in epithelial cells promotes M2 macrophage infiltration and thus disease progression. Zileuton is the only anti-leukotriene inhibitor approved by the U.S. Food and Drug Administration (FDA). The use of Zileuton improves lung function in children and adults with mild to severe asthma, highlighting the potential of blocking this pathway to treat chronic inflammation [44, 45]. In this study, treatment with Zileuton was effective in reducing ICC tumor volume and inhibiting M2 macrophage infiltration, improving the suppressive immune microenvironment. Moreover, co-treatment with PLX3397 can greatly improve the therapeutic effect.

This study also has many shortcomings. First, ALOX5 may have the function of activating other pathways in addition to its metabolic enzyme itself. ALOX5 may affect the secretion of some cytokines or chemokines in ICC cells. Although we confirmed that its downstream metabolite LTB4 is the dominant factor affecting M2 macrophage migration, other factors should not be ignored. Subsequently, we will screen the factors that may affect M2 macrophages by high-throughput sequencing to further improve the viewpoint of this study. Secondly, this study lacks clinical relevance studies with large clinical samples. The clinical relevance of ALOX5 was not explored in depth in this study, and correlation analysis is often difficult due to the difficulty of ICC sample acquisition. However, related work is ongoing, and the establishment of ICC high-throughput sequencing database will help to improve the clinical background of this gene. Finally, the use of animal models is too limited. Due to the lack of mature commercial murine cell lines for ICC, the in vitro experiments in this study were not performed with primary cells. Also, we chose a subcutaneous tumor model rather than an orthotopic tumor model. There is a need to construct more reliable in vivo ICC models, starting with primary cells in vivo, to help improve the level of evidence for research.

## Conclusions

In conclusion, this study systematically studied the mechanism of ALOX5/LTB4/TAM axis regulating ICC progression in the development of ICC, and provided a new idea for clinical diagnosis and treatment of ICC by elucidating the new mechanism of ICC lipid metabolism reprogramming remodeling immune microenvironment.

### Supplementary Information

**Additional file 1: Figure S1.** The infiltration of TAM in ICC and normal tissues was analyzed by immunohistochemistry. (a) Immunohistochemical images showed the expression of CD68 in ICC tissue and adjacent normal tissue. (b) Immunohistochemical images demonstrated the expression of CD163 in ICC tissue and adjacent normal tissue. (c) Immunohistochemical images showed the expression of CD206 in ICC tissue and adjacent normal tissue.

**Additional file 2.** Comparison of TAM infiltration abundance between dense and non-dense epithelial areas in 12 ICC tissues (Red: EPCAM; Green: CD163).

**Additional file 3.** Top 60 degs in 7 paired ICC tissues.

**Additional file 4.** Comparison of TAM infiltration abundance in regions with high or low ALOX5 expression level in ICC tissues (Red: ALOX5; Green: CD163).

**Additional file 5.** RNA-seq result of Zileuton vs NC (gene expression).

**Additional file 6.** RNA-seq result of Zileuton vs NC (differentially expressed genes).

## Data Availability

The data could be downloaded at EMBL-EBI database (https://www.ebi.ac.uk/), GEO database (https://www.ncbi.nlm.nih.gov/geo/) and TCGA database (https://portal.gdc.cancer.gov), and the code used during the current study are available from the corresponding author on reasonable request.

## Abbreviations

GEO: Gene Expression Omnibus
NC: Negative control
TCGA: The Cancer Genome Atlas
TAM: Tumor-associated Macrophages
LTB4: Leukotriene B4
GO: Gene Onotology
KEGG: Kyoto Encyclopedia of Genes and Genomes
siRNA: SmallinterferingRNA
GSEA: Gene Set Enrichment Analysis
IL-4: Interleukin-4
IL-13: Interleukin-13
