# Supplementary material for: ALOX5 acts as a key role in regulating the immune microenvironment in intrahepatic cholangiocarcinoma, recruiting tumor-associated macrophages through PI3K pathway
Source: J Transl Med. 2023 Dec 20;21:923. doi: 10.1186/s12967-023-04804-1 (PMC10734103; doi:10.1186/s12967-023-04804-1)
Supplement: Supplementary file 1 — Additional file 1: Figure S1. The infiltration of TAM in ICC and normal tissues was analyzed by immunohistochemistry. (a) Immunohistochemical images showed the expression of CD68 in ICC tissue and adjacent normal tissue. (b) Immunohistochemical images demonstrated the expression of CD163 in ICC tissue and adjacent normal tissue. (c) Immunohistochemical images showed the expression of CD206 in ICC tissue and adjacent normal tissue. [file 12967_2023_4804_MOESM1_ESM.docx]

**ALOX5 Acts as A Key Role in Regulating the Immune Microenvironment in Intrahepatic Cholangiocarcinoma, Recruiting Tumor-associated Macrophages through PI3K Pathway**

**Jialu Chen^1,3^, Yue Tang^1,3^, Delong Qin^1,3^, Xiaopeng Yu^1,3^, Huanjun Tong^1,3^, Chengwei Tang^1^, *Zhaohui Tang^1,2,3^**

**^1^ Department of General Surgery, Xinhua Hospital, Shanghai Jiao Tong University, School of Medicine, Shanghai 200092, China**

**^2^ Department of Blood Transfusion, Xinhua Hospital, Shanghai Jiao Tong University, School of Medicine, Shanghai 200092, China**

**^3^ Shanghai Key Laboratory of Biliary Tract Disease Research, Xinhua Hospital, Shanghai Jiao Tong University School of Medicine, Shanghai 200092, China.**

**Corresponding author: Zhaohui Tang**

**tzh1236@163.com**


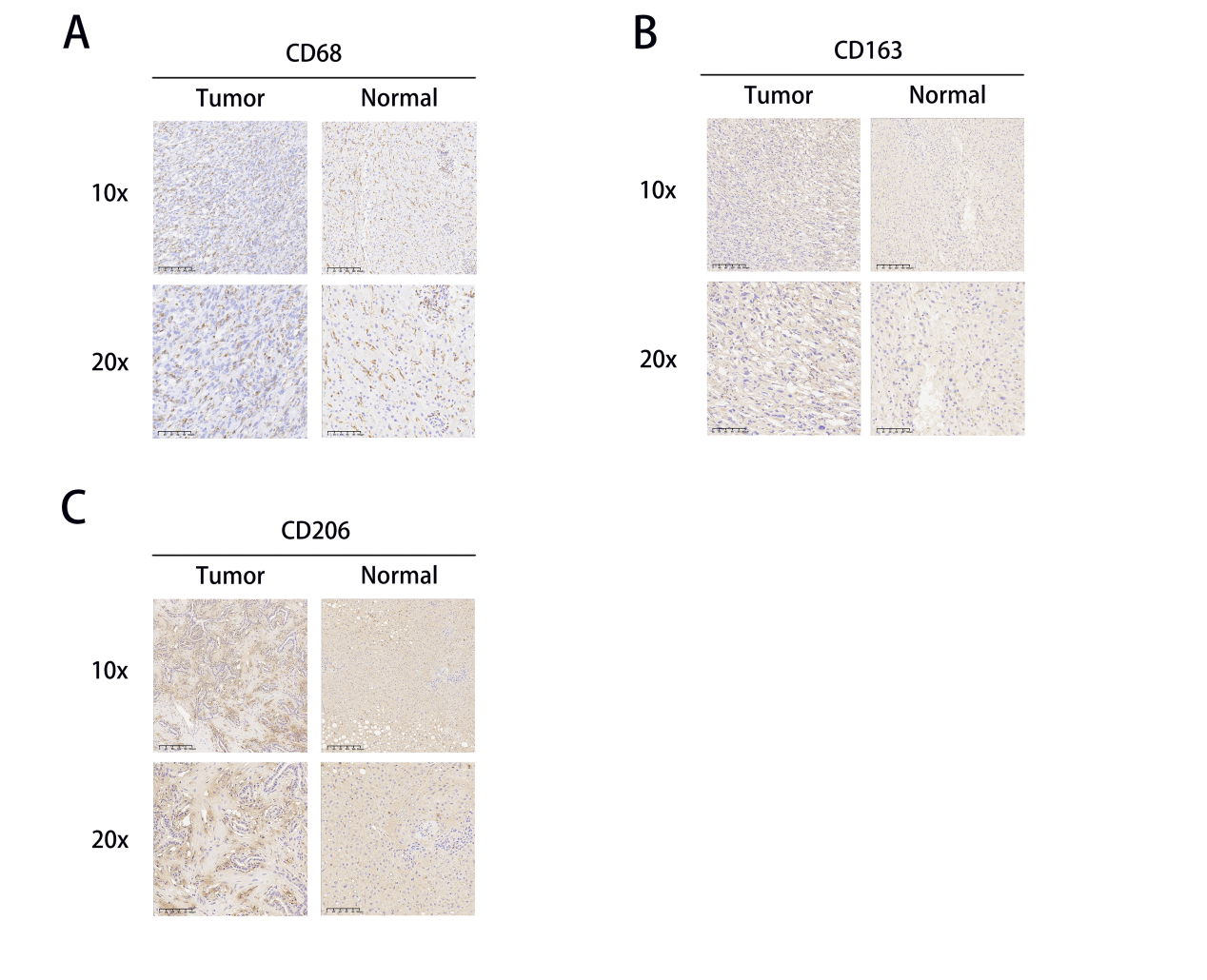


Supplementay Fig.S1. The infiltration of TAM in ICC and normal tissues was analyzed by immunohistochemistry.

1. Immunohistochemical images showed the expression of CD68 in ICC tissue and adjacent normal tissue. (b) Immunohistochemical images demonstrated the expression of CD163 in ICC tissue and adjacent normal tissue. (c) Immunohistochemical images showed the expression of CD206 in ICC tissue and adjacent normal tissue.
