# Supplementary material for: ALOX5 acts as a key role in regulating the immune microenvironment in intrahepatic cholangiocarcinoma, recruiting tumor-associated macrophages through PI3K pathway
Source: J Transl Med. 2023 Dec 20;21:923. doi: 10.1186/s12967-023-04804-1 (PMC10734103; doi:10.1186/s12967-023-04804-1)
Supplement: Supplementary file 4 — Additional file 4. Comparison of TAM infiltration abundance in regions with high or low ALOX5 expression level in ICC tissues (Red: ALOX5; Green: CD163). [file 12967_2023_4804_MOESM4_ESM.docx]

**ALOX5 Acts as A Key Role in Regulating the Immune Microenvironment in Intrahepatic Cholangiocarcinoma, Recruiting Tumor-associated Macrophages through PI3K Pathway**

**Jialu Chen^1,3^, Yue Tang^1,3^, Delong Qin^1,3^, Xiaopeng Yu^1,3^, Huanjun Tong^1,3^, Chengwei Tang^1^, *Zhaohui Tang^1,2,3^**

**^1^ Department of General Surgery, Xinhua Hospital, Shanghai Jiao Tong University, School of Medicine, Shanghai 200092, China**

**^2^ Department of Blood Transfusion, Xinhua Hospital, Shanghai Jiao Tong University, School of Medicine, Shanghai 200092, China**

**^3^ Shanghai Key Laboratory of Biliary Tract Disease Research, Xinhua Hospital, Shanghai Jiao Tong University School of Medicine, Shanghai 200092, China.**

**Corresponding author: Zhaohui Tang**

**tzh1236@163.com**


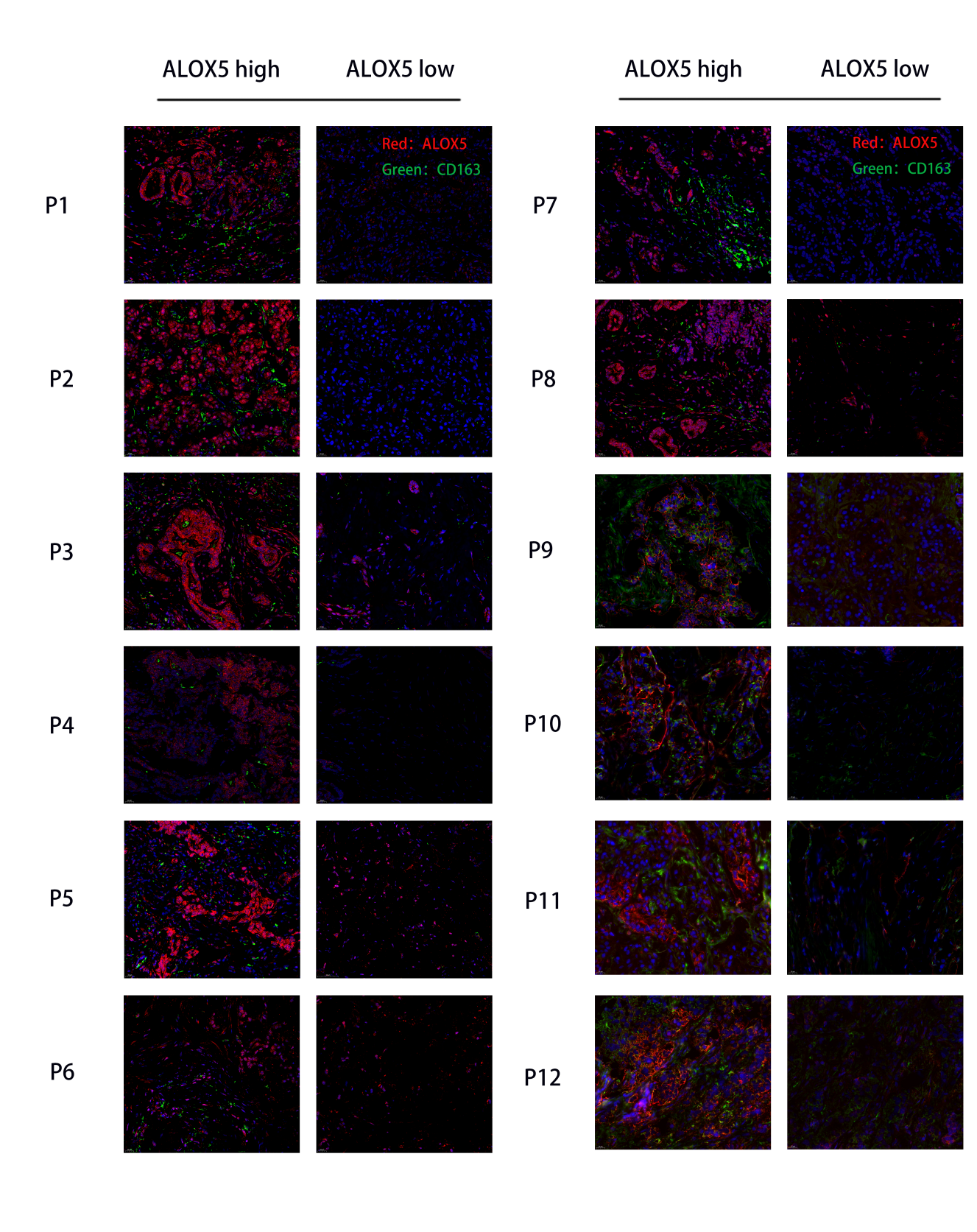


Supplementay Fig.S3. Comparison of TAM infiltration abundance in regions with high or low ALOX5 expression level in ICC tissues. (Red: ALOX5; Green: CD163)
